# Editorial: Cerebellum-related learning and psychiatric diseases

**DOI:** 10.3389/fncel.2023.1132286

**Published:** 2023-02-13

**Authors:** Gen Ohtsuki, Taegon Kim, Peng Sun, Yongjun Chen, Egidio Ugo D'Angelo

**Affiliations:** ^1^Department of Drug Discovery Medicine, Kyoto University Graduate School of Medicine, Kyoto, Japan; ^2^Brain Science Institute, Korea Institute of Science and Technology, Seoul, Republic of Korea; ^3^Department of Pharmacology, Shandong University of Traditional Chinese Medicine, Jinan, China; ^4^Research Institute for Acupuncture and Moxibustion, Shangdong University of Traditional Chinese Medicine, Jinan, China; ^5^Department of Brain and Behavioral Sciences, University of Pavia, Pavia, Italy

**Keywords:** cerebellum, learning, psychiatric disorders, molecules, immune cells, plasticity, functional connectivity

The cerebellum—little brain in Latin—is one of the most studied brain regions concerning cellular physiology, circuit, and plasticity. The well-organized neural circuit of the cerebellum has defined its functionality for motor coordination and motor learning. Recent motivations and challenges for the regulation and its interaction with other brain regions, such as the midbrain, thalamus, hypothalamus, hippocampus, and prefrontal cortex, opened a new set of questions regarding the role of the cerebellum in higher-order cognitive function and dysfunction. In this Research Topic, we called many innovative scientists to gather the current knowledge about cerebellar learning and mental illness in the range of the established and the hypothesized. We accepted studies in human patients with psychiatric disorders associated with cerebellar dysfunction. Additionally, we included studies in animal models that report the analysis of molecular mechanisms in the cerebellum underlying potential pathology. Those studies gave us much insight into the mechanisms for cerebellum-related learning and psychiatric disorders.

Pierre Flourens found involvement of the cerebellum in motor coordination in the nineteen century with the observation that cerebellectomized pigeons had impairments in posture and locomotion. Since the discovery, the cerebellum has received much interest from neurophysiologists in terms of motor coordination and its learning. The discovery of synaptic plasticity (e.g., long-term depression of parallel fiber synapses) was widely accepted as the basis of cerebellar learning and memory (Ito, 2008). But now, the various forms of cerebellar plasticity have been identified ubiquitously in synaptic connection and intrinsic excitability of neurons (Hansel et al., 2001; Ohtsuki et al., 2020). Those forms of cerebellar plasticity may also contribute to spatiotemporal integration and error reduction. However, it is yet unclear how the spatiotemporal coordinate system is expressed in the brain against the space, and how it is learned and perceived in the cerebellum.

While most studies for long-term plasticity performed recording from the neurons in slice sections of the cerebellum, Lu et al.  demonstrated that facial stimulation induced mossy fiber-granule cell (MF-GrC) LTP in the mouse granule cell layer *in vivo*. The induction of MF-GrC LTP was dependent on GluR2A subunit-containing NMDA receptors but not those containing GluNR2B or GluNR2C/D subunits. MF-GrC LTP was accompanied by a decrease in the paired-pulse ratio and *via* nitric oxide production, suggesting an increase in presynaptic release. MF-GrC LTP by sensory stimulation may play some roles in cerebellar adaptation and learning.

The relevance of the cerebellar circuit in disease should not be surprising. Many symptoms of motor dysfunction in patients with ataxia and Parkinson's disease may arise from cerebellar dysfunction. Meanwhile, it was just a couple of decades ago since researchers recognized that the cerebellum is involved in higher cognition as well as motor function (Schmahmann and Sherman, 1998). The cerebellar dysfunction is regarded to lead to cognitive dysfunction in patients with neurodevelopmental disorders: autism spectrum disorders (ASD), developmental disorders (including ADHD), schizophrenia, and mood disorders. A learning disability may result from impairments of cerebellar function.

Fragile X syndrome (FXS) is caused solely by a mutation of the FMR1 gene on the X chromosome. Intellectual disability and behavioral symptoms like anxiety and ADHD are manifest in patients with FXS. The FMR1 gene encodes FMRP (Fragile X Messenger Ribonucleoprotein), which is expressed in the whole brain, including the cerebellum, and is involved in synaptic plasticity. Oe et al. showed that cytoplasmic polyadenylation element-binding protein 1 (CPEB1), an RNA-binding protein, controls the intracellular localization of Fmr1 mRNA in neurons and post-transcriptionally regulates Fmr1 expression. Also, CPEB1 involvement in mitochondrial functions and localization of heat shock protein family A member 9 was observed in a cellular model of FXS. Their finding of regulation of Fmr1 expression by CPEB1 will contribute to the elucidation of the pathogenesis of FXS.

As aforementioned, impairments of synaptic transmission, and synaptic plasticity of Purkinje cells in the cerebellum lead to motor dyscoordination. In contrast, the plasticity of intrinsic excitability also became known to contribute to cerebellar adaptation learning (Schonewille et al., 2010; Ryu et al., 2017). Remarkably, the inflammatory mediators from microglia modulate both synaptic efficacy and intrinsic excitability of Purkinje cells, which disrupts animal behaviors (Yamamoto et al., 2019). Brain inflammation is highly related to an anomaly of animal behaviors and the emergence of psychiatric disorders and neurodegenerative diseases. Reactive microglia scavenge debris in the brain and phagocytose degenerated neurons and synapses in a disorder model (Shi et al., 2009). Therefore, brain inflammation and the resultant dysfunction of neurons could be the key concepts for understanding cerebellum-related psychiatric disorders.

Hikosaka et al. reviewed the induction of plasticity by various inflammatory mediators, such as cytokines and chemokines, and summarized the different forms of plasticity in neuronal activity. Most immune cells in the brain are microglia, which release inflammatory mediators after activation and induce forms of plasticity in neurons. Such immune-triggered plasticity was reported repeatedly in different types of neurons across the central nervous system and spinal cord, suggesting biological significance and inflammatory disease relevance. We speculate that a distinct population of immune cells emerges in different locations of the psychiatric-disorder brains and leads to neurophysiological impairment and degeneration. Nevertheless, further investigations of the brains of human patients, as well as disorder models, are required because mental illnesses ultimately cannot be solved in animal models (Insel, 2010).

In humans, non-invasive studies by brain imaging using magnetic resonance imaging (MRI) are often carried out. Feng et al. explored the functional connectivity of cerebellar networks of first-episode schizophrenia patients. As expected, the functional connectivity in first-episode schizophrenia patients was lower than healthy controls. The functional connectivity was positively and negatively correlated with the severity of positive and negative symptoms, respectively, indicating that network dynamics in the cerebellum were closely linked with the polarity of the symptom. Meanwhile, Ding et al. studied patients with major depressive disorder (MDD) associated with gastrointestinal symptoms. The authors showed cerebellar default mode network (DMN) in gastrointestinal symptoms correlated with MDD severity, reflecting the higher frequency of gastrointestinal symptoms and the greater depression severity. Resting-state functional MRI and the cerebellar seed-based functional connectivity maps revealed that anterior cerebellar DMN was enhanced in patients with gastrointestinal symptoms. The right Crus I - right superior temporal gyrus connectivity correlated with the severity of gastrointestinal symptoms.

Overall, this Research Topic gathered interesting studies from the molecular mechanism, modulation of neural activity, and cognitive impairments. As for the involvement of the cerebellum in psychiatric disorders, the novel type of neural plasticity by immune cells potentially plays a crucial role, and the induction of cerebellar plasticity may be disturbed in the cerebellum as pathophysiological impairments.

## Author contributions

GO edited the first draft of the manuscript and revised the manuscript. GO, TK, PS, YC, and EU reviewed and finalized the manuscript. All authors have read and approved the final version of the article for publication.
